# Physiological and Transcriptional Responses to Phosphorus Deficiency and Glucose-6-Phosphate Supplementation in *Neopyropia yezoensis*

**DOI:** 10.3390/ijms252312894

**Published:** 2024-11-30

**Authors:** Yujiao Chen, Senhao He, Yinghao Wang, Chuanming Hu, Weitao Cheng, Lingjie Zhou, Nanjing Ji, Haihong Chen, Xin Shen

**Affiliations:** 1 Jiangsu Key Laboratory of Marine Bioresources and Environment, Jiangsu Key Laboratory of Marine Biotechnology, Jiangsu Ocean University, Lianyungang 222005, China; chenyujiao0828@126.com (Y.C.); 2023210104@jou.edu.cn (S.H.); m19516919160@163.com (Y.W.); cwt18109635480@163.com (W.C.); chenhhhailey@163.com (H.C.); 2 Co-Innovation Center of Jiangsu Marine Bio-Industry Technology, Jiangsu Ocean University, Lianyungang 222005, China; 3 Jiangsu Marine Fisheries Research Institute, Nantong 226007, China; hucharming@163.com; 4 Scripps Institution of Oceanography, University of California, San Diego, CA 92093, USA; liz047@ucsd.edu

**Keywords:** *Neopyropia yezoensis*, phosphorus deficiency, glucose-6-phosphate, physiological, transcriptome

## Abstract

*Neopyropia yezoensis*, a marine red algae species, has significant economic and ecological value. However, phosphorus (P) deficiency has emerged as a growing concern in many cultivation regions, negatively impacting its growth. To adapt to P deficiency, algae have evolved various strategies, including using dissolved organic phosphorus (DOP) sources to sustain growth. Despite its prevalence as a form of DOP, the utilization mechanism of glucose-6-phosphate (G6P) by *N. yezoensis* remains unclear. In this study, the physiological and transcriptional responses of *N. yezoensis* to P deficiency and G6P supplementation were examined. The results demonstrated that prolonged P deficiency significantly inhibited the growth of *N. yezoensis* and had a negative impact on physiological indicators such as photosynthetic pigments and antioxidant enzyme activity. However, G6P treatment gradually alleviated these adverse effects over time. Both P deficiency and G6P treatment were associated with increased expression of genes involved in signal transduction and P starvation responses while concurrently downregulating genes related to photosynthesis and antioxidant defenses. In contrast, the suppression of gene expression was less significant under G6P treatment. This study elucidates the adaptive strategies of *N. yezoensis* in response to P deficiency and clarifies the regulatory pathways involved in G6P utilization, providing novel insights into its P nutrient acquisition and metabolic regulation.

## 1. Introduction

*Neopyropia yezoensis*, a member of the Rhodophyta, is an economically important seaweed widely distributed along the coasts of East Asia [1]. Rich in proteins, vitamins, and minerals, this species is extensively used in the food and pharmaceutical industries [2]. Moreover, the cultivation of *N. yezoensis* holds significant economic and ecological value, contributing to marine ecosystem restoration and sustainability [3]. However, rising sea temperatures, ocean acidification, and increased ultraviolet (UV) radiation due to global climate change pose serious threats to the habitat of economic algae, leading to reductions in yield and quality [4]. Moreover, imbalances in nitrogen (N) and phosphorus (P) levels in aquatic environments negatively affect the growth of these species. Perini and Bracken [5] reported that the interaction between limiting nutrients, such as N and P, affects the growth and function of *Fucus vesiculosus*, with N availability influencing P uptake. Similarly, Kim et al. [6] suggested that P deficiency is a key factor limiting the growth of *N. yezoensis* in the Nakdong River Estuary.

Phosphorus is a key nutrient that regulates marine primary productivity and ecosystem health [7]. It plays a vital role in cellular structure formation, energy metabolism, and regulating physiological processes in organisms [8]. Ocean-dissolved P is classified into soluble reactive phosphorus (SRP) and soluble non-reactive phosphorus (SNP). SRP primarily consists of dissolved inorganic phosphorus (DIP), while SNP is mainly composed of dissolved organic phosphorus (DOP) [9]. Phosphorus deficiency, driven by factors such as water column stratification and anthropogenic activities, has been reported in regions like the Atlantic Ocean, the central Mediterranean Sea, and the northern South China Sea [10,11,12,13,14,15]. In coastal areas, P deficiency can severely impact the aquaculture of economically significant algae, reducing growth and yield in species such as *Gracilaria lemaneiformis* and *Agarophyton vermiculophyllum* [16,17,18]. Moreover, P deficiency can alter algal community structures, promoting the proliferation of harmful algal species adapted to low P conditions, thereby threatening the sustainability of aquaculture environments [19]. To cope with P deficiency, algae have evolved adaptive mechanisms, including the use of microbial enzymatic reactions and the secretion of phosphatases to convert DOP into bioavailable inorganic phosphate [20,21,22]. In addition, algae can store polyphosphate granules intracellularly to meet metabolic demands during P scarcity [23,24]. Moreover, studies have shown that the SPX (SYG/PHO81/XPR1) and phosphate starvation response 1 (PHR1) gene families play a crucial role in the regulation of P sensing and transport, and the regulation of their expression helps plants optimize P utilization and enhance stress resistance during P deficiency [25,26]. Extracellular alkaline phosphatase (AP) production has been studied in five species of marine algae, with evidence suggesting its involvement in the utilization of DOP glycerophosphate [27]. However, the mechanisms underlying DOP utilization in *N. yezoensis*, particularly the molecular pathways of DOP assimilation and responses to P deficiency, remain largely unexplored.

This study employed G6P as a source of DOP and established low concentrations of inorganic phosphate levels. Through a combination of physiological analyses, including enzyme activity assays, and transcriptomic analysis, the research investigated the responses of *N. yezoensis* to P deficiency and G6P supplementation. The findings elucidated the distinctive role of G6P in P supply, providing insights into its function in P acquisition strategies. It enhanced our understanding of how *N. yezoensis* copes with P deficiency, underscoring the critical role of G6P in its adaptive response.

## 2. Results

### 2.1. Changes in N. yezoensis Growth and DIP Concentration in Medium Under Different Culture Conditions

**Figure 1:**
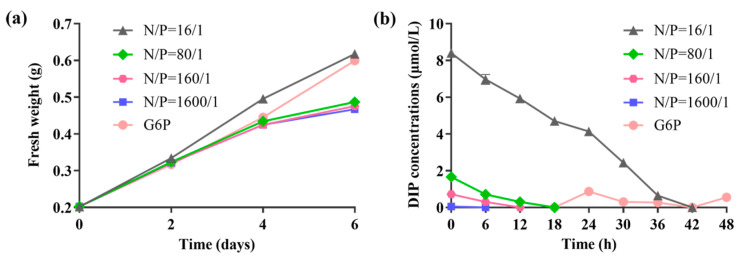
Changes in fresh weight of *N. yezoensis* thalli and DIP concentration in the culture medium under different N-to-P ratios and a G6P-treated group. (**a**) Fresh weight changes of thalli under varying groups. (**b**) Changes in DIP concentration in the culture medium. The DIP concentrations for the four groups with N/P ratios of 16:1, 80/1, 160/1, and 1600/1 were 8.58 μM, 1.72 μM, 0.86 μM, and 0.09 μM, respectively. The dissolved inorganic nitrogen (DIN) concentration for each group was set at 137.25 μM, and the G6P concentration was 21 μM.

During the 6-day cultivation period, the fresh weight of *N. yezoensis* increased across all experimental groups. The most rapid growth was observed in the group with an N/P ratio of 16/1, while the G6P group had slower initial growth that accelerated later in the cultivation period. In contrast, the P_deficiency groups (N/P ratios of 80/1, 160/1, and 1600/1) showed consistently slower growth, with only minor differences observed among these groups (Figure 1a). The DIP concentration progressively declined in all treatment groups over time. Specifically, DIP levels in the 16/1, 80/1, 160/1, and 1600/1 N/P ratio groups barely detectable at 42, 18, 12, and 6 h, respectively. The G6P group maintained detectable DIP levels from 18 to 48 h, albeit at minimal concentrations (Figure 1b).

### 2.2. Changes in N and P Content in N. yezoensis Thalli Under Different Culture Conditions

**Figure 2:**
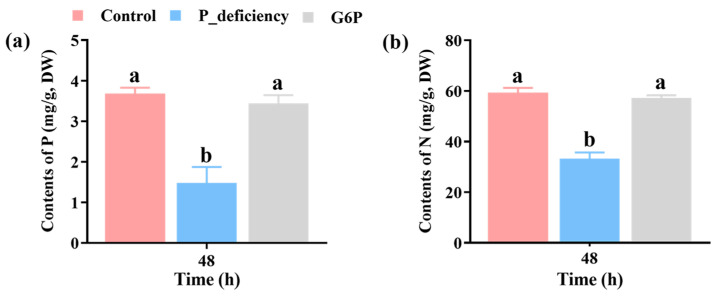
Effects of P deficiency and G6P as a P source on phosphorus (P) and nitrogen (N) content in *N. yezoensis* thalli. (**a**) P content. (**b**) N content. Different lowercase letters (a, b) indicate significant differences between treatments at the same time point (*p* < 0.05).

Results indicated that, relative to the Control group, the P content in the P_deficiency group decreased by 59.8%, while the N content decreased by 44.0% (*p* < 0.05). In contrast, the N and P contents in the G6P group did not exhibit significant differences from those in the Control group (*p* > 0.05) and were significantly higher than those in the P_deficiency group (*p* < 0.05) (Figure 2a,b). These results suggest that P deficiency impairs N and P accumulation in *N. yezoensis*, whereas using G6P as a P source can effectively alleviate this effect.

### 2.3. Changes in Photosynthetic Pigment Content Under Different Culture Conditions

**Figure 3:**
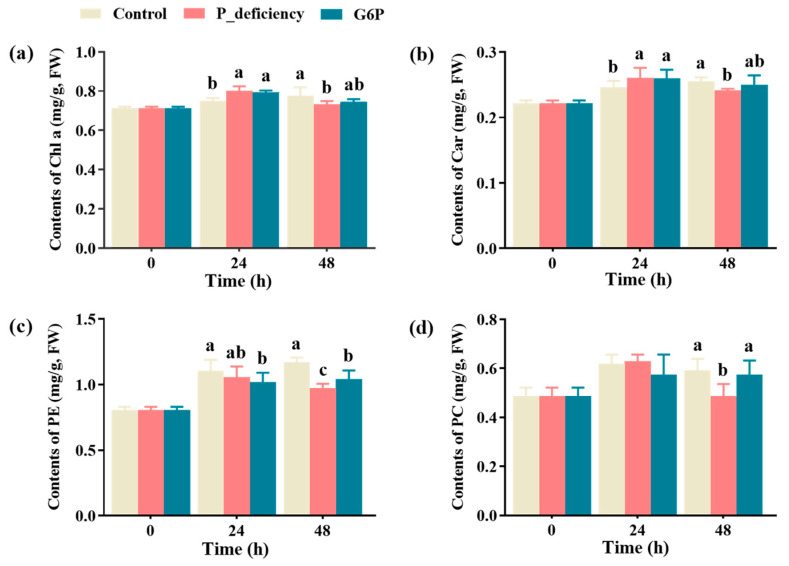
Effects of P deficiency and G6P as a P source on photosynthetic pigments in *N. yezoensis* thalli. (**a**) Chlorophyll a (Chl a) content. (**b**) Carotenoid (Car) content. (**c**) Phycoerythrin (PE) content. (**d**) Phycocyanin (PC) content. Different lowercase letters (a, b, c) indicate significant differences between treatments at the same time point (*p* < 0.05).

Measurements of photosynthetic pigments in the thalli revealed that, at 24 h, chlorophyll a and carotenoid levels were significantly higher in both the P_deficiency and G6P groups compared to the Control (*p* < 0.05). By 48 h, these levels had notably decreased in the P_deficiency group relative to the Control (*p* < 0.05), with no significant change in the G6P group (*p* > 0.05) (Figure 3a,b). Phycoerythrin levels at 48 h were lower in the G6P and P_deficiency groups than in the Control (*p* < 0.05) (Figure 3c). In addition, at 48 h, the contents of phycoerythrin and phycocyanin in the G6P group were significantly higher than those in the P_deficiency group (*p* < 0.05) (Figure 3c,d). These results suggest that prolonged P deficiency may inhibit pigment synthesis, while G6P supplementation mitigates these effects, stabilizing pigment levels during later stages.

### 2.4. Changes in Malondialdehyde (MDA) and Hydrogen Peroxide (H_2_O_2_) Levels, Along with Antioxidant Enzyme Activity in Thalli Under Different Culture Conditions

**Figure 4:**
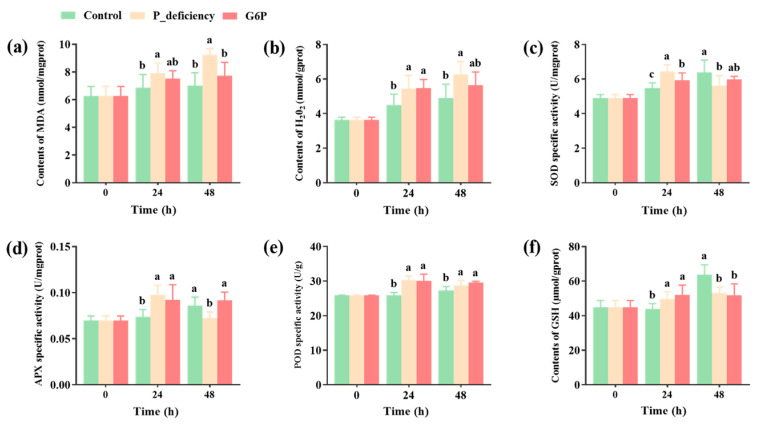
Effects of P deficiency and G6P as a P source on MDA and H_2_O_2_ levels, as well as antioxidant enzyme activities in *N. yezoensis* thalli. (**a**) Malondialdehyde (MDA) content. (**b**) Hydrogen peroxide (H_2_O_2_) content. (**c**) Superoxide dismutase (SOD) activity. (**d**) Ascorbate peroxidase (APX) activity. (**e**) Peroxidase (POD) activity. (**f**) Glutathione (GSH) content. Different lowercase letters (a, b, c) indicate significant differences between treatments at the same time point (*p* < 0.05).

Measurements of MDA and H_2_O_2_ levels, along with those of antioxidant enzymes, indicated a significant increase in H_2_O_2_ and MDA levels in the P_deficiency group under prolonged stress compared to the Control (*p* < 0.05). In contrast, in the G6P group, these levels were observed to be reduced relative to the P_deficiency group at 48 h (Figure 4a,b). SOD and APX activities in the P_deficiency group showed an initial increase followed by a decline, whereas, in the G6P group, enzyme activities increased early without significant changes thereafter (Figure 4c,d). At both 24 and 48 h, POD activity in the P_deficiency and G6P groups exceeded that of the Control (*p* < 0.05), although a slight decline was observed in both groups by 48 h compared to the 24-h levels (*p* > 0.05) (Figure 4e). Furthermore, GSH content in both the P_deficiency and G6P groups was significantly higher than in the Control at 24 h but decreased significantly by 48 h (*p* < 0.05) (Figure 4f). These findings suggest that P deficiency and G6P supplementation considerably influence MDA and H_2_O_2_ levels, as well as antioxidant enzyme activities in *N. yezoensis*, with the effects of P deficiency being more significant.

### 2.5. Identification of Differentially Expressed Genes (DEGs)

**Figure 5:**
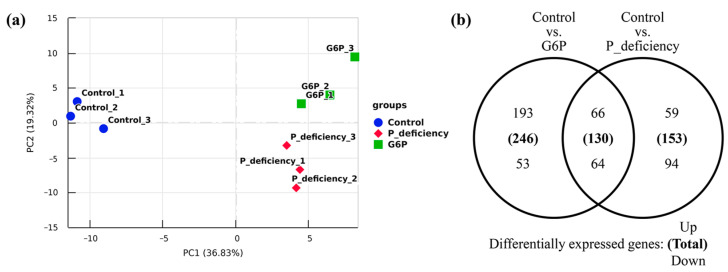
Identification of differentially expressed genes (DEGs) in *N. yezoensis* under P deficiency and G6P as a P source. (**a**) Principal component analysis (PCA) plot showing gene expression values (FPKM) for all samples. The x-axis represents the first principal component, and the y-axis represents the second. (**b**) Venn diagram of DEGs comparing P deficiency vs. Control and G6P vs. Control groups. The overlap between the circles represents shared DEGs between both groups. Bold numbers indicate total DEGs, with numbers above and below indicating upregulated and downregulated genes, respectively.

RNA–seq analysis was conducted on *N. yezoensis* thalli after 48 h of culture. Following data filtration, over 93% of the raw reads were retained as clean reads, with Q30 quality scores ranging from 92.51% to 93.31% across all samples (Table S1). Clean reads showed a mapping rate exceeding 88% to the reference genome for all samples (Table S2). Principal component analysis (PCA) indicated clear separation among treatment groups and tight clustering within groups, reflecting significant differences in gene expression patterns (Figure 5a). These results confirm the high quality of the sequencing data, supporting its suitability for further analyses.

In the P_deficiency vs. Control comparison, 283 DEGs were identified, consisting of 125 upregulated and 158 downregulated genes. In the G6P vs. Control comparison, 376 DEGs were detected, with 259 genes upregulated and 117 downregulated. Moreover, 130 DEGs were shared between the P_deficiency vs. Control and G6P vs. Control comparisons, including 66 upregulated and 64 downregulated genes (Figure 5b). However, it is important to note that the identified DEGs have not undergone quantitative real-time PCR verification. This limitation should be addressed in future research.

### 2.6. Enrichment Analysis of Differentially Expressed Genes

KEGG pathway enrichment analysis was used to assess the functional annotations of DEGs (Figure S1). In the P_deficiency vs. Control comparison, eight pathways, including “carbon fixation in photosynthetic organisms”, were significantly enriched (*P*adj < 0.05). In contrast, only three pathways were significantly enriched for the G6P vs. Control comparison using a more relaxed significance threshold (*P*adj < 0.1). These results indicate that P deficiency has a more significant impact on the metabolic pathways of *N. yezoensis*, whereas the impact of G6P as a P source appears to be relatively modest.

**Figure 6:**
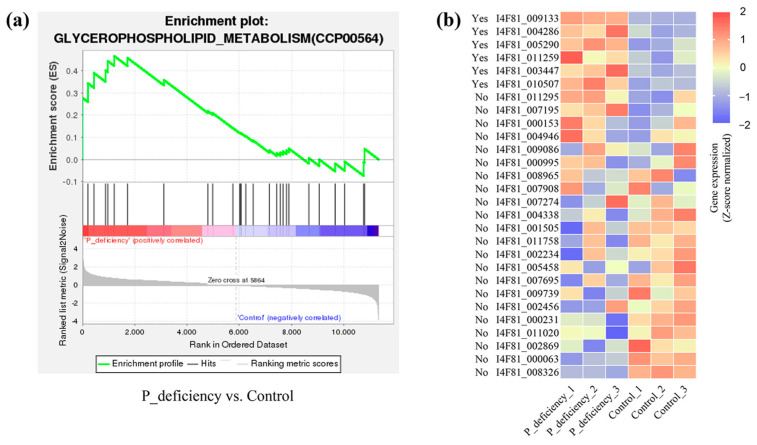
GSEA of pathways significantly enriched in the P deficiency phenotype and heatmap of related genes. (**a**) Glycerophospholipid metabolism pathway enrichment analysis. The peak in the line chart represents the enrichment score (ES) of the gene set. The green curve represents the trend of the ES across the ranked gene list. The vertical black lines (Hits) indicate the positions of individual genes within the gene set along the ranked list. In the color bar, the red region represents genes positively correlated with “P_deficiency”, while the blue region represents genes positively correlated with “Control”. The gray shaded area reflects the distribution of ranking metric scores. (**b**) Heatmap showing the relative expression levels (FPKM) of genes enriched in the glycerophospholipid metabolism pathway. “Yes” indicates that the gene contributes to the ES score, while “No” indicates that it does not. Gene annotations include I4F81_009133: glycerophosphocholine phosphodiesterase 1 (gde1); I4F81_005290: phosphatidate phosphatase (Lpin2); I4F81_003447: glycerol-3-phosphate dehydrogenase 1 (GLY1); and I4F81_004286, I4F81_011259, and I4F81_010507, all annotated with PF02230: phospholipase/carboxylesterase.

Gene set enrichment analysis (GSEA) was performed using KEGG gene sets, identifying 10 significantly upregulated gene sets each in the P_deficiency and G6P groups, respectively (Table S3) [28]. Specifically, in the P_deficiency group, the glycerophospholipid metabolism pathway was the only significantly enriched pathway, meeting the criteria of |NES| > 1, NOM *p*-val < 0.05, and FDR q-val < 0.25 (Figure 6a). Six genes, including glycerophosphocholine phosphodiesterase 1 (*gde1*), glycerol-3-phosphate dehydrogenase 1 (*GLY1*), and phosphatidate phosphatase (*Lpin2*), contributed significantly to the enrichment score within this pathway, which is essential for phospholipid synthesis and metabolic regulation (Figure 6b). Overall, these findings suggest that *N. yezoensis* modulates glycerophospholipid metabolism to optimize P utilization, thereby maintaining membrane stability and functionality under P deficiency conditions.

### 2.7. Differential Expression of Genes Related to N and P Metabolism, Signal Transduction, Photosynthesis, and Antioxidant Mechanisms

**Figure 7:**
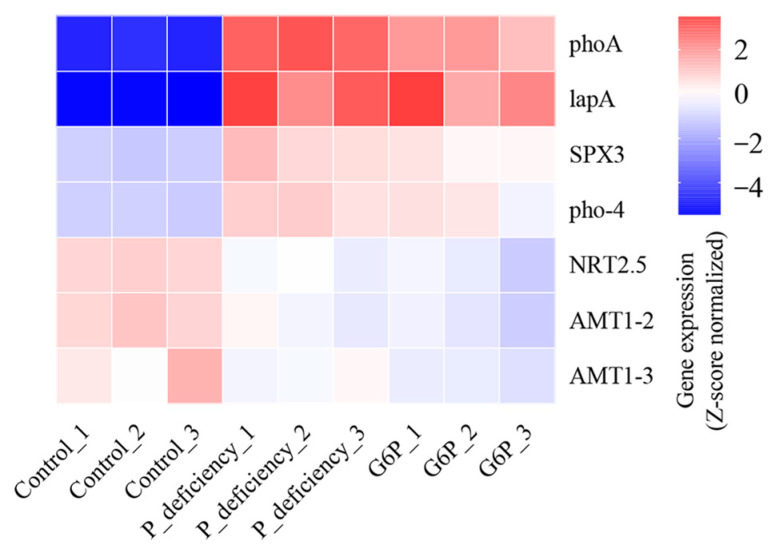
Heatmap of differentially expressed genes (DEGs) associated with phosphorus and nitrogen metabolism. LapA and phoA: alkaline phosphatase; SPX3: SPX domain-containing protein 3; pho-4: phosphate permease 4; NRT2.5: nitrate transporter 2.5; AMT1-2 and AMT1-3: ammonium transporter. The heatmap displays the relative expression of DEGs, with the color scale representing the Row Z-score derived from FPKM values.

As shown in Figure 7, genes involved in P metabolism showed significant upregulation under both P deficiency and G6P treatment. The expression of alkaline phosphatases, *LapA*, and *phoA*, increased significantly, with *LapA* rising by 890.91-fold under P deficiency and 736.36-fold with G6P treatment. The SPX domain-containing protein 3 (*SPX3*), a phosphate sensor, was upregulated 5.20-fold under P deficiency and 3.28-fold with G6P. Furthermore, the phosphate transporter gene, phosphate permease 4 (*pho-4*), had increased expression across both conditions (Figure 7). These results suggest that *N. yezoensis* effectively modulates P-related gene expression to adapt to low-P environments and enhance G6P utilization. In contrast, genes related to N metabolism showed downregulation under P deficiency and G6P treatment. Interestingly, expression levels of ammonium transporters (*AMT1-2* and *AMT1-3*), as well as the nitrate transporter (*NRT2.5*), were significantly downregulated (Figure 7). This downregulation may reflect a regulatory mechanism to maintain cellular N-P balance under P deficiency conditions.

**Figure 8:**
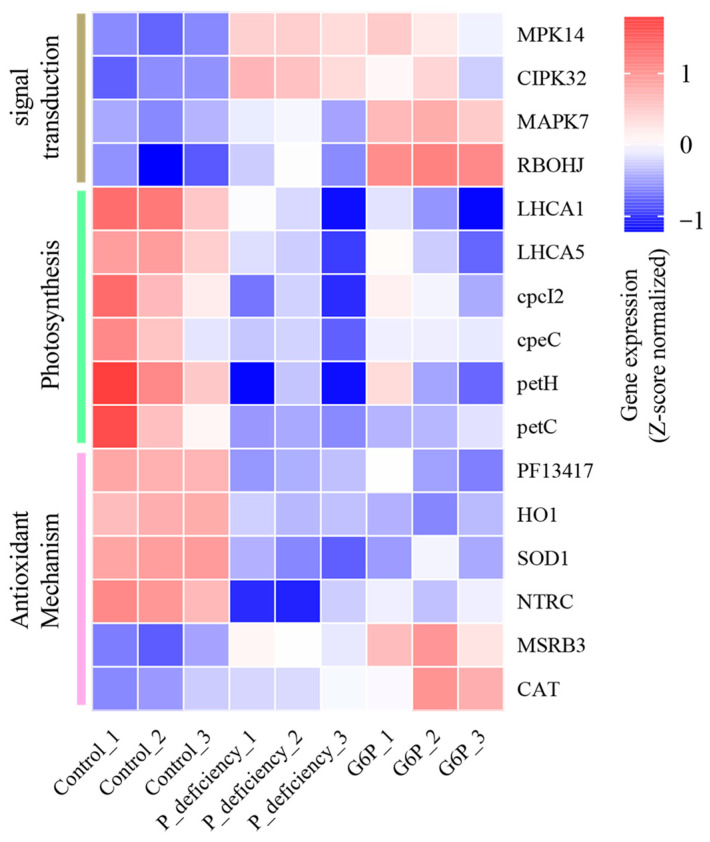
Heatmap of differentially expressed genes (DEGs) associated with signal transduction, photosynthesis, and antioxidant mechanisms. MPK14: mitogen-activated protein kinase 14; CIPK32: CBL-interacting protein kinase 32; MAPK7: mitogen-activated protein kinase 7; RBOHJ: respiratory burst oxidase homolog J; LHCA1 and LHCA5: light-harvesting complex I chlorophyll a/b-binding protein; cpcI2: phycocyanin-associated linker protein; cpeC: the phycoerythrin-associated linker protein; petH: ferredoxin-NADP+ reductase; petC: cytochrome b6/f complex iron-sulfur subunit; PF13417: glutathione S-transferase N-terminal domain proteins; HO1: heme oxygenase 1; SOD1: Cu, Zn superoxide dismutase; NTRC: NADPH-dependent thioredoxin reductase C; MSRB3: methionine sulfoxide reductase B3; CAT: catalase. The heatmap displays the relative expression of DEGs related to these functions, with the color scale representing the Row Z-score derived from FPKM values.

The mitogen-activated protein kinase (MAPK) and CBL-interacting protein kinase (CIPK) signaling pathways are involved in regulating plant responses to environmental stress. Under P deficiency, *N. yezoensis* showed significant upregulation of mitogen-activated protein kinase 14 (*MPK14*) and CBL-interacting protein kinase 32 (*CIPK32*) by 2.18-fold and 2.77-fold, respectively (Figure 8). In the G6P treatment group, mitogen-activated protein kinase 7 (*MAPK7*) showed a 2.18-fold increase, while respiratory burst oxidase homolog J (*RBOHJ*), a key player in the ROS signaling pathway, was upregulated by 4.08-fold (Figure 8). These findings indicate that *N. yezoensis* activates specific signaling pathways to optimize P utilization, particularly from G6P, and adapt to P-deficient conditions.

In the KEGG enrichment analysis, several photosynthesis-related pathways were significantly downregulated under P deficiency (Figure S1). The genes encoding light-harvesting complex I chlorophyll a/b-binding proteins, such as *LHCA1* and *LHCA5*, were downregulated by 2.78-fold and 2.30-fold, respectively. Similarly, genes related to algal photosynthetic pigment-protein complexes, including the phycocyanin-associated linker protein (*cpcI2*) and the phycoerythrin-associated linker protein (*cpeC*), were downregulated by 2.77-fold and 2.04-fold, respectively. Furthermore, genes involved in photosynthetic electron transfer, such as ferredoxin-NADP+ reductase (*petH*, 4.25-fold downregulation) and cytochrome b6/f complex iron-sulfur subunit (*petC*, 3.10-fold downregulation), were also significantly downregulated (Figure 8). Under G6P treatment, the same set of genes also showed downregulation: *LHCA1* (3.15-fold), *LHCA5* (2.12-fold), *cpcI2* (1.93-fold), *cpeC* (1.64-fold), *petH* (2.77-fold), and *petC* (2.60-fold) (Figure 8). However, the magnitude of downregulation was less significant compared to P deficiency. These findings indicate that P deficiency more significantly impacts photosynthesis-related gene expression in *N. yezoensis*, compared to the effects observed when G6P is used.

Regarding antioxidant defense mechanisms, P deficiency significantly downregulated several genes, including NADPH-dependent thioredoxin reductase C (*NTRC*), Cu, Zn superoxide dismutase (*SOD1*), and heme oxygenase 1 (*HO1*), as well as genes encoding glutathione S-transferase N-terminal domain proteins (PF13417) (Figure 8). This suggests that prolonged P deficiency may compromise the antioxidant defense system in *N. yezoensis*. In contrast, under G6P treatment, there was a reduction in the downregulation of these genes, accompanied by upregulation of catalase (*CAT*) and methionine sulfoxide reductase B3 (*MSRB3*) (Figure 8). This indicates that G6P, as an organic P source, may help augment P availability and partially mitigate the adverse effects on the antioxidant defense system.

## 3. Discussion

### 3.1. Signal Transduction

In plants, MAPKs, CIPKs, and ROS play key roles in signal transduction and response to environmental stress. The MAPK pathway, for example, is involved in regulating cell cycle progression, development, and stress resistance through phosphorylation cascades [29]. In this study, *N. yezoensis* showed upregulation of *MPK14* and *CIPK32* expression under P deficiency (Figure 8). Previous research on *Arabidopsis thaliana* has shown that *CIPK1* acts as a regulatory factor in P starvation signaling, influencing cellular P homeostasis [30]. These findings suggest that *MPK14* and *CIPK32* may similarly contribute to the P deficiency response in *N. yezoensis*, playing a role in its adaptation to environmental stress. Under G6P treatment, *MAPK7* and *RBOHJ* expression was upregulated in *N. yezoensis* (Figure 8). The RBOH family, which consists of NADPH oxidases, catalyzes ROS production, serving as key signaling molecules that regulate growth, development, pathogen defense, and environmental stress responses [31]. The observed upregulation of *RBOHJ* in this study indicates that *N. yezoensis* may regulate ROS production via *RBOHJ* activation during G6P assimilation, enabling adaptation to physiological changes and environmental challenges. Previous studies have shown that the interaction among MAPK, CIPK, and ROS forms a complex signaling network that regulates plant responses to stress [32,33]. For instance, Qiao et al. [34] reported that in *Monoraphidium* sp., ROS, calcium (Ca^2^⁺), and MAPK interact to regulate biomass production and lipid biosynthesis under combined salt and H_2_O_2_ stress. Similarly, in *A. thaliana*, RBOHs have been shown to play a central role in the Ca^2^⁺-ROS signaling network activated by phosphorylation during stress adaptation [35]. In the current study, the expression of MAPK, CIPK, and ROS-related genes in *N. yezoensis* was significantly upregulated under both P deficiency and G6P treatment, suggesting a potential interactive mechanism among these pathways. This interaction may collaboratively regulate the physiological and metabolic activities of *N. yezoensis*, supporting its growth and adaptation under low P conditions or when utilizing organic P sources such as G6P. These findings underscore the complexity of the signaling regulatory mechanisms in *N. yezoensis*.

### 3.2. Phosphorus and Nitrogen Metabolism

Plants respond to P starvation by regulating the expression of phosphate-starvation-responsive (PSR) genes, including alkaline phosphatases (APs) and SPX family genes [36]. APs facilitate the breakdown of organic P compounds, releasing inorganic P essential for growth and metabolism in marine algae [37]. Genes encoding APs are commonly upregulated under P starvation [38,39]. In this study, *N. yezoensis* showed significant upregulation of *LapA* and *phoA* under P deficiency (Figure 7). The *pho-4* gene, which encodes a phosphate permease, plays a crucial role in the transmembrane transport of phosphate, with expression increasing under low-P conditions to improve phosphate uptake efficiency [40,41]. Moreover, the SPX domain acts as a regulator in phosphate sensing and signaling, modulating cellular responses to phosphate availability [42]. SPX domain-related genes, identified in rice and *A. thaliana*, show complex regulatory behaviors under phosphate starvation [43,44]. Consistent with these findings, *pho-4* and *SPX3* were also upregulated in *N. yezoensis* in response to P deficiency (Figure 7). Despite the adaptive upregulation of P-related genes, the P content in *N. yezoensis* thalli remained significantly reduced under P deficiency due to the limited availability of external P (Figure 2a). Under G6P treatment, the expression of *LapA*, *phoA*, *pho-4*, and *SPX3* was also upregulated, although to a lesser extent compared to P deficiency conditions (Figure 7). The P content of the thalli under G6P treatment did not differ significantly from that of the control group (Figure 2a), suggesting that while the assimilation rate of G6P may be relatively slow, *N. yezoensis* can still optimize P metabolic pathways through the upregulation of relevant genes. This response enhances the acquisition of inorganic P and enables effective utilization of G6P, maintaining stable P content in the thalli.

N and P metabolism in plants are closely linked, forming a regulatory mechanism that supports growth and adaptation to environmental changes [45]. For instance, the PHR1-NIGT1-NRT2.1 transcriptional cascade regulates the uptake and metabolism of phosphate (PO_4_^3−^) and nitrate (NO_3_^−^), where P starvation induces *NIGT1* expression, thereby reducing NO_3_^−^ uptake [46]. Similarly, signaling pathways such as OsNRT1.1B-OsSPX4-OsPHR2 and AtNRT1.1-PHO2-HRS1 are essential for coordinating N and P metabolism in plants [47,48,49]. In this study, mRNA levels of the N transporter genes *NRT2.5*, *AMT1-2*, and *AMT1-3* were downregulated under P deficiency (Figure 7). Correspondingly, the N content in *N. yezoensis* thalli was significantly reduced under P deficiency (Figure 2b), consistent with findings in corn and tobacco where P limitation impairs N uptake [50,51]. In contrast, the G6P-treated group did not show a significant change in N content compared to the Control group (Figure 2b), although N transporter genes were still downregulated (Figure 7). This suggests that *N. yezoensis* can utilize G6P to maintain N metabolism, preventing a significant decline in N content. However, due to the relatively slow assimilation of G6P, the algal cells may still perceive signals resembling P deficiency, leading to the downregulation of N transporter gene expression.

### 3.3. Glycerophospholipid Metabolism

Glycerophospholipids, a subclass of phospholipids, are essential components of cell and organelle membranes in plants, contributing to membrane fluidity, stability, and functionality [52,53]. In this study, GSEA revealed that the glycerophospholipid metabolism pathway was significantly upregulated under P deficiency (Figure 6a,b). Key genes in this pathway, such as *gde1* and *GLY1*, are involved in intracellular signaling and lipid metabolism [54,55]. Moreover, three genes encoding the PF02230: phospholipase/Carboxylesterase domain contribute to cellular regulation, membrane lipid remodeling, and lipid degradation [56]. Plants experiencing environmental stresses like drought, salinity, and P deficiency can modulate glycerophospholipid composition and metabolism to maintain membrane structure and function, thereby enhancing stress tolerance [57,58,59]. Similarly, phytoplankton increases the production of alternative lipids (e.g., sulfolipids) under P deficiency to sustain membrane stability and function [59,60,61]. Based on these studies, it is inferred that *N. yezoensis* may employ a similar mechanism, adjusting membrane lipid composition to preserve cellular function during P deficiency. Moreover, previous studies have indicated that phospholipid degradation gene expression is upregulated in SPX mutants, suggesting that SPX proteins may act as negative regulators of phospholipid degradation [62]. In the present study, the upregulation of the *SPX3* gene implies that *N. yezoensis* may regulate phospholipid metabolism through an SPX-mediated feedback mechanism, limiting excessive phospholipid degradation to maintain stable intracellular phospholipid levels. Overall, these findings suggest that *N. yezoensis* adapts to P deficiency by modulating the glycerophospholipid metabolic pathway and *SPX* gene expression, thereby optimizing membrane composition and functionality.

### 3.4. Photosynthesis

Photosynthetic pigments, such as chlorophyll a, carotenoids, phycoerythrin, and phycocyanin, are essential for capturing light energy and converting it into chemical energy to drive photosynthetic reactions, supporting energy and carbon metabolism in plants and algae [63]. Previous studies have shown that P deficiency significantly reduces photosynthetic pigment content, as observed in the diatom *Thalassiosira weissflogii* [64]. Similarly, this study found a significant decrease in pigment levels in *N. yezoensis* under P deficiency (Figure 3). Although G6P supplementation did not entirely prevent pigment reduction, the decrease was less severe compared to P deficiency alone (Figure 3), suggesting that G6P may partially mitigate the adverse effects of P deficiency on photosynthesis.

KEGG pathway enrichment analysis revealed significant downregulation of photosynthesis-related pathways, including carbon fixation in photosynthetic organisms, under P deficiency (Figure S1). Specifically, genes encoding antenna complex proteins (e.g., *LHCA1*, *LHCA5*, *cpcI2*, and *cpeC*) and components of the electron transport chain (e.g., *petH* and *petC*) were significantly downregulated (Figure 8). Similar responses have been observed in crops like rice and soybean, where photosynthesis-related genes are significantly downregulated during P deficiency [65,66]. The disruption of the electron transport chain due to P deficiency may elevate ROS levels, leading to oxidative stress [67,68]. However, some studies suggest that the downregulation of photosynthesis-related genes may serve as an adaptive response to minimize ROS production by reducing the activity of the photosynthetic machinery [65]. In this study, the downregulation of photosynthesis-related genes in *N. yezoensis* under P deficiency may represent an adaptive mechanism to sustain basic metabolic functions and mitigate the damaging effects of nutrient stress. Under G6P treatment, when the statistical threshold was adjusted to *P*adj < 0.1, only two photosynthesis-related pathways showed significant downregulation, and the extent of gene suppression was less pronounced than under P deficiency alone. The combined physiological and molecular data indicate that P deficiency significantly impairs photosynthesis in *N. yezoensis*, but G6P assimilation can partially alleviate the inhibitory effects, suggesting a potential role of G6P in supporting photosynthetic activity during P deficiency.

### 3.5. Antioxidant System

Plants exposed to environmental stresses, such as low P conditions and high temperatures, can produce excessive ROS, leading to oxidative stress that impairs growth and development. To counteract the damaging effects of ROS, plants have developed a sophisticated antioxidant system with various enzymes and regulatory genes [69]. In this study, a significant increase in H_2_O_2_ and MDA levels was observed under P deficiency compared to the Control, indicating a significant oxidative stress response (Figure 4a,b). This was accompanied by a rise in the activities of SOD, APX, and POD at 24 h, followed by a decline at 48 h (Figure 4c,d,e). These findings are consistent with previous studies demonstrating that prolonged P deficiency can reduce the activities of key antioxidant enzymes, including SOD, POD, and catalase (CAT) [70,71]. It suggest that while antioxidant enzymes play a crucial role in mitigating ROS accumulation initially, their effectiveness is reduced with extended or severe stress, potentially due to the depletion of cellular resources [72]. Moreover, GSH levels in the Control group were significantly higher than in the P_deficiency and G6P groups after 48 h (Figure 4f). Similarly, genes associated with antioxidant defense, such as *HO1*, *SOD1*, and *NTRC*, along with genes encoding glutathione S-transferase domains, were downregulated during prolonged stress (Figure 8). This suggests that *N. yezoensis* activates its antioxidant enzyme system during short-term P deficiency as an immediate stress response. However, as stress persists, the synthesis of antioxidant enzymes becomes impaired, leading to weakened defense mechanisms and exacerbating the negative impact of oxidative stress. Under G6P treatment, the activities of antioxidant enzymes like SOD, APX, and POD increased after 24 h but did not show a significant increase after 48 h (Figure 4c,d,e), consistent with the observed changes in H_2_O_2_ and MDA levels (Figure 4a,b). Moreover, although some antioxidant genes, such as *HO1*, *SOD1*, and *NTRC*, were downregulated, others, like *MSRB3* and *CAT*, showed upregulation (Figure 8). The gradual assimilation of G6P may have alleviated P starvation in *N. yezoensis*, thereby mitigating oxidative stress and allowing the regulation of specific antioxidant genes to facilitate partial recovery of the antioxidant defense system. These findings indicate that under P deficiency, *N. yezoensis* experiences significant oxidative stress, with its antioxidant enzyme system initially activated but eventually compromised under extended stress. G6P treatment appears to ameliorate some of the adverse effects by supporting the modulation of antioxidant gene expression, thus helping the algae manage oxidative stress more effectively.

## 4. Materials and Methods

### 4.1. Experimental Materials and Culture Conditions

Thalli of *N. yezoensis* were collected from aquaculture rafts in Yancheng, Jiangsu Province, China (119.97° E, 34.48° N) in December 2023. Healthy thalli, measuring 5–8 cm in length and free of visible damage or disease, were selected for this study. Cultures were maintained in enriched seawater artificial water (ESAW) medium with modified N and P concentrations [73]. The culture conditions were set at 8 ± 0.5 °C, with a light intensity of 40 μmol m^−2^ s^−1^ and a 12:12 h light/dark cycle. Aeration was continuously provided throughout the experiment.

### 4.2. N. yezoensis Different P Treatments

The experiment included five treatment groups: four groups with varying DIP concentrations and one group treated with G6P. Each group had three biological replicates. NaH_2_PO_4_·H_2_O and NaNO_3_ served as the P and N sources, respectively. Based on the Redfield ratio, the N-to-P ratios in the DIP groups were set at 16:1, 80:1, 160:1, and 1600:1, corresponding to DIP concentrations of 8.58 μM, 1.72 μM, 0.86 μM, and 0.09 μM, respectively [74]. Following the ESAW medium protocol [73], PO_4_^3−^ was replaced with 21 µM G6P, while the concentration of dissolved inorganic nitrogen (DIN) was uniformly maintained at 137.25 μM across all experimental treatments.

Algal cultures were grown in 500 mL of artificial seawater in spherical glass culture bottles, each containing 0.2 g of fresh algal biomass. The algae were acclimated for 48 h at a 16:1 N-to-P ratio before starting experimental treatments. During the six-day experiment, the culture medium was replenished every 48 h, and the fresh weight of the algal biomass was recorded bi-daily. DIP concentrations in the culture medium were measured every 6 h using the molybdenum blue colorimetric method.

### 4.3. Investigation of the Physiological Responses of N. yezoensis Under Varying Culture Conditions

The experiment involved three treatment groups: Control, P_deficiency, and G6P, each consisting of three biological replicates. P concentrations in the culture medium were set at 10 μM for the Control, 1 μM for P_deficiency, and 21 μM for the G6P group, while N concentration was maintained at a constant 160 μM across all groups. In each experimental setup, 0.4 g of fresh algal biomass was introduced into 1000 mL of artificial seawater per bottle. After a 48-h acclimation period, algae were allocated to the respective treatment groups. During the initial 48 h of cultivation, no water exchange was performed. Samples were collected at 0, 24, and 48 h to measure physiological parameters.

For chlorophyll and carotenoid analysis, 0.05 g of fresh *N. yezoensis* thalli were homogenized in 1 mL of 100% methanol using a Scientz-48L cryogenic high-throughput tissue grinder (Scientz, Ningbo, China) at 1500 RPM and 4 °C for 10 min. An additional 4 mL of 100% methanol was added, and the mixture was incubated overnight in the dark at 4 °C. Following extraction, the samples were centrifuged, and the supernatant was collected for absorbance measurements at 470 nm, 653 nm, and 666 nm using a microplate photometer (ThermoFisher Scientific, Waltham, MA, USA). Chlorophyll a and carotenoid concentrations were calculated based on the method described by Wellburn [75].

To measure phycoerythrin and phycocyanin, 0.05 g of fresh *N. yezoensis* thalli were homogenized in 1 mL of phosphate-buffered saline (PBS; 0.1 mol/L, pH 6.8) using a Scientz-48L cryogenic high-throughput tissue grinder (Scientz, Ningbo, China) at 1500 RPM and 4 °C for 10 min. Following the addition of 4 mL of PBS, the mixture underwent four freeze-thaw cycles. The samples were then centrifuged, and the supernatant was collected for absorbance measurements at 455 nm, 564 nm, 592 nm, 618 nm, and 645 nm. The concentrations of phycoerythrin and phycocyanin were calculated using the method described by Beer and Eshel [76].

The contents of hydrogen peroxide (H_2_O_2_), malondialdehyde (MDA), and reduced glutathione (GSH), as well as the activities of superoxide dismutase (SOD), ascorbate peroxidase (APX), and peroxidase (POD), were assessed using commercial kits from Nanjing Jiancheng Bioengineering Institute (Nanjing, China) according to the manufacturer’s protocols.

To determine the N and P contents in the algae, fresh samples were collected after 48 h of culture and analyzed at Shanghai Youxuan Biotechnology Co., Ltd (Shanghai, China). The N content in the thalli was quantified using the sulfuric acid-mixed accelerator-distillation method (NY/T 2419-2013), while P content was measured using the molybdenum-antimony anti-colorimetric method (NY/T 2421-2013).

### 4.4. Transcriptome Sequencing

Fresh thalli of *N. yezoensis*, cultured for 48 h, were ground in liquid nitrogen to obtain a fine powder. Approximately 50–100 mg of the powdered sample was transferred into a 1.5 mL centrifuge tube, followed by adding 1 mL of TRIzol reagent (Molecular Research Center, Cincinnati, OH, USA). The mixture was homogenized thoroughly for RNA extraction. The quality of the extracted RNA was assessed using a Bioanalyzer 2100 (Agilent Technologies, Palo Alto, CA, USA), and the RNA integrity number (RIN) was calculated to confirm the integrity and quality of the samples. A cDNA library was constructed using the NEB library preparation protocol. Sequencing was performed by Novogene (Beijing, China) on the Illumina HiSeq 2500 platform, implementing reference-based transcriptome sequencing with the *N. yezoensis* reference genome ASM982973V1 (GenBank assembly: GCA_009829735.1). Differential gene expression analysis (|log2(FoldChange)| ≥ 1, Padj ≤ 0.05) was conducted using the R package DESeq2. Data quality assessment and further analysis were conducted on the Novomagic cloud platform (https://magic.novogene.com/ accessed on 15 April 2024). The RNA–seq data have been deposited in the National Center for Biotechnology Information (NCBI) database under BioProject accession number PRJNA1161186.

## 5. Conclusions

This study provides insights into the physiological and transcriptional responses of *N. yezoensis* under P deficiency and G6P treatment. The findings demonstrate that P deficiency significantly inhibits the growth of *N. yezoensis*, reducing the accumulation of N and P within the thalli. Further, this deficiency results in decreased photosynthetic pigment levels, significant downregulation of photosynthesis-related gene expression, persistent elevation of ROS, and a decline in antioxidant enzyme activity alongside the downregulation of antioxidant genes. These observations suggest that prolonged P deficiency not only disrupts photosynthesis but also induces oxidative stress. Despite these adverse effects, *N. yezoensis* employs various adaptive mechanisms, such as regulation of signal transduction, activation of the antioxidant system, optimization of P metabolism, and regulation of glycerophospholipid metabolic pathway, to cope with P deficiency. The study also highlights that G6P, a representative DOP compound, can alleviate some of the negative impacts of P deficiency through its gradual assimilation, supporting cellular functions and partially mitigating stress-related damage. Overall, these findings deepen our understanding of how *N. yezoensis* responds to P deficiency and lay the groundwork for future studies aimed at improving the resilience of marine algae to nutrient stress and their ability to utilize various P sources.

## Data Availability

In this study, all data generated are included in this article. Further enquiries can be directed to the corresponding author.

## Supplementary Materials

The following supporting information can be downloaded at: https://www.mdpi.com/article/10.3390/ijms252312894/s1.
