# Supplementary material for: Physiological and Transcriptional Responses to Phosphorus Deficiency and Glucose-6-Phosphate Supplementation in Neopyropia yezoensis
Source: Int J Mol Sci. 2024 Nov 30;25(23):12894. doi: 10.3390/ijms252312894 (PMC11641120; doi:10.3390/ijms252312894)
Supplement: Supplementary file 1 [file ijms-25-12894-s001.zip › figure caption.pdf]

Figure S1: KEGG enrichment analysis scatter plot of DEGs in *N. yezoensis* under P deficiency and organic P conditions. (a) KEGG enrichment analysis scatter plot for the P_deficiency vs. Control group. (b) KEGG enrichment analysis scatter plot for the G6P vs. Control group.
