# Supplementary figures and images for: Physiological and Transcriptional Responses to Phosphorus Deficiency and Glucose-6-Phosphate Supplementation in Neopyropia yezoensis
Source: Int J Mol Sci. 2024 Nov 30;25(23):12894. doi: 10.3390/ijms252312894 (PMC11641120; doi:10.3390/ijms252312894)

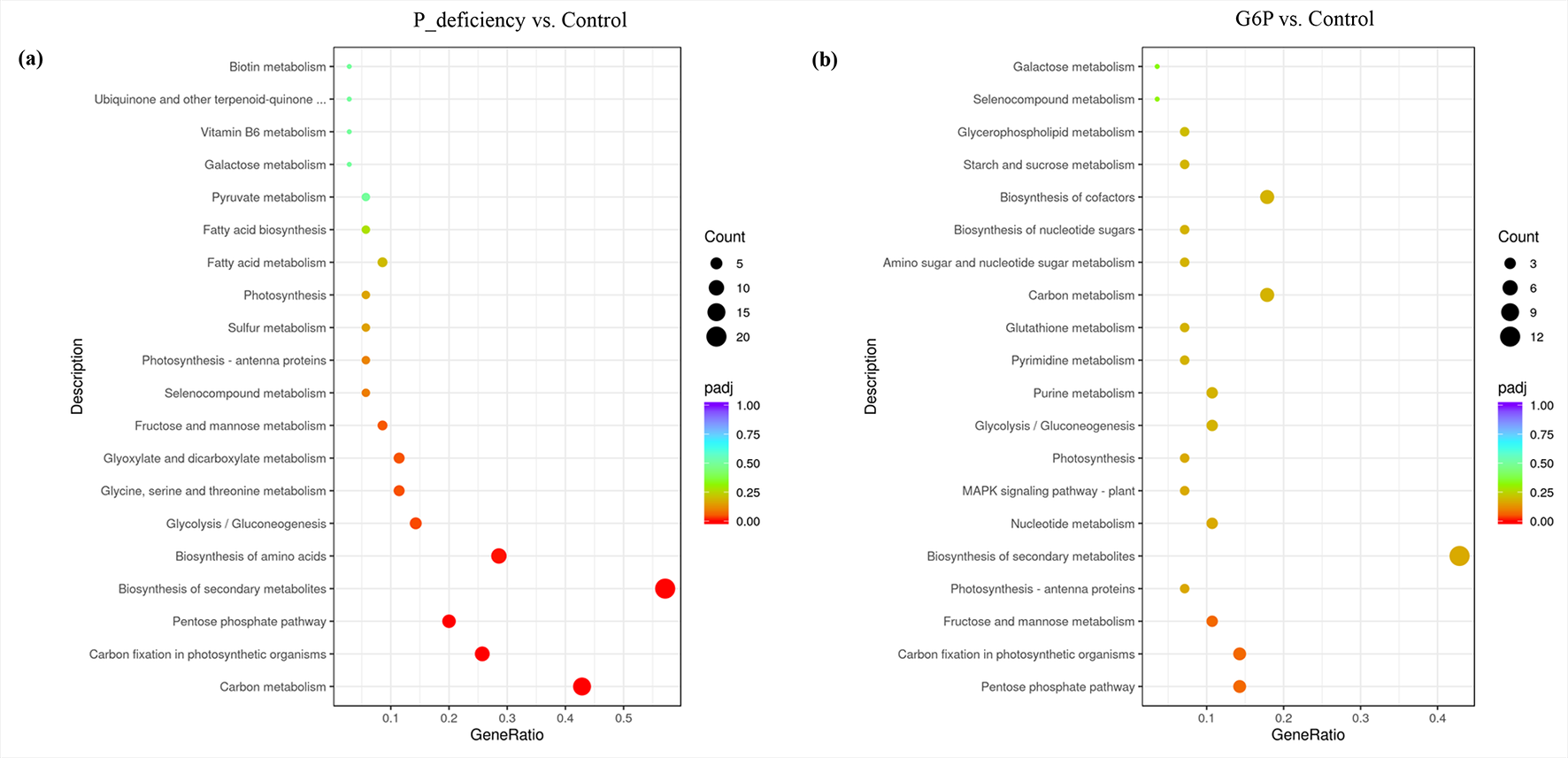

Supplement: Supplementary file 1 [file ijms-25-12894-s001.zip › Figure S1.tif]
